# Endoscopic ultrasound-directed transenteric retrograde cholangiopancreatography with cholangioscopy-guided argon plasma coagulation for removal of uncovered metal stents

**DOI:** 10.1055/a-2832-6121

**Published:** 2026-03-25

**Authors:** Marco Spadaccini, Giacomo Marcozzi, Miriana Mercurio, Valeria Poletti, Matteo Colombo, Alessandro Fugazza, Alessandro Repici

**Affiliations:** 1 9268 Department of Gastroenterology, IRCCS Humanitas Research Hospital, Rozzano, Milan, Italy; 2 437807 Department of Biomedical Sciences, Humanitas University, Pieve Emanuele, Milan, Italy

The management of biliary uncovered self-expandable metallic stents (uSEMSs) complicated by tissue ingrowth represents a major technical challenge, particularly in patients with surgically altered anatomy 1 2 . For this reason, uSEMSs should not be used in benign disease as failed stent removal may expose patients with otherwise non-malignant conditions to life-threatening complications, including the need for major surgery or liver failure due to secondary sclerosing cholangitis.

This video demonstrates how appropriate awareness and the combined use of technical and technological innovations enabled the successful endoscopic management of a complex case. A 64-year-old patient with prior total gastrectomy for gastric adenocarcinoma and Roux-en-Y reconstruction had been treated percutaneously for biliary stones several years earlier, with the placement of two biliary uSEMSs. As expected, the patient subsequently required repeated percutaneous interventions for recurrent cholangitis.

When referred to our center for acute cholangitis in August 2025, the patient underwent complex entero-endoscopic retrograde cholangiopancreatography performed using push enteroscopy with a colonoscope, which revealed multiple stones impacted within the stent and extensive diffuse tissue ingrowth. After complete stone clearance, a fully covered SEMS was placed using a stent-in-stent technique. To facilitate future access to the biliary limb, an endoscopic ultrasound (EUS)-guided entero-enterostomy was also performed. One month later, EUS-directed transenteric retrograde cholangiopancreatography was carried out through the lumen-apposing metal stent 3 .

**Fig. 1:**
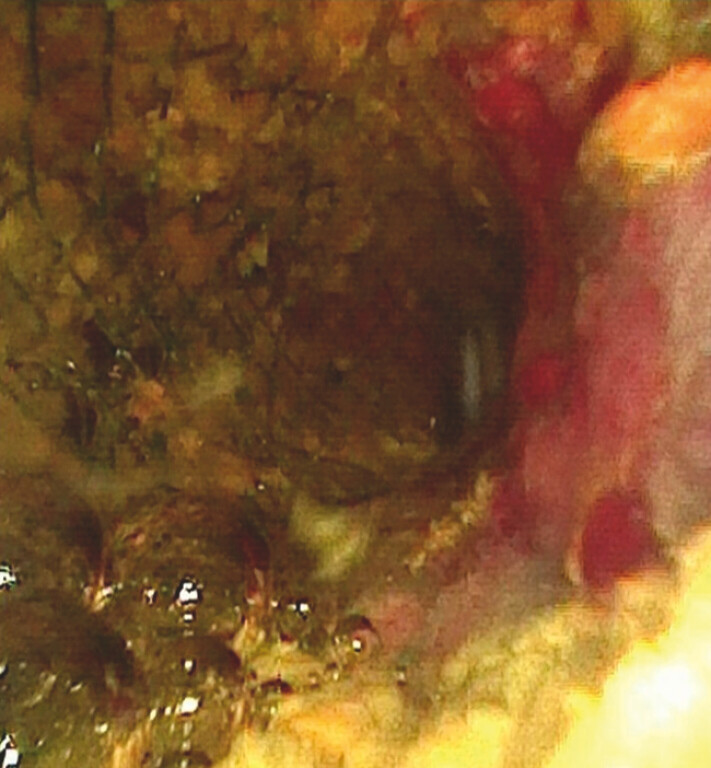
A cholangioscopic view of proximal stent ingrowth.

**Fig. 2:**
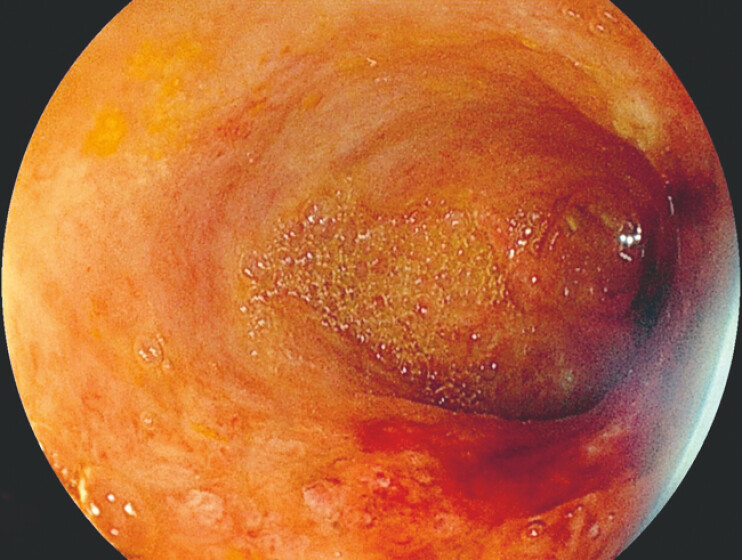
Direct visualization of the common bile duct after stent removal.

Using a single-use gastroscope with a 4.2-mm working channel, biliary access was achieved with a novel large-caliber cholangioscope 4 . Cholangioscopy confirmed dense ingrowth at both proximal and distal ends of the two uncovered stents ( Fig. 1 ). The 2-mm working channel allowed passage of a 1.5-mm argon plasma coagulation (APC) probe, enabling controlled intraductal ablation of exuberant tissue under direct visualization. APC was applied in pulsed mode at 30–40 W with a gas flow rate of 0.8–1.0 L/min under carbon dioxide insufflation. Broad-spectrum intravenous antibiotic prophylaxis was administered peri-procedurally. Complete tissue ablation ultimately permitted the successful removal of the uSEMS more than 4 years after implantation using foreign body forceps, with restoration of bile duct patency ( Video 1 ). No procedure-related adverse events occurred ( Fig. 2 ). The patient showed rapid clinical improvement and was discharged after a short hospital stay without early complications. During follow-up, no further episodes of cholangitis were observed.

An EUS-directed entero-enterostomy, combined with the use of a single-use gastroscope enabled large-caliber cholangioscopy and APC-guided intraductal ablation, ultimately allowing complete stent removal and restoration of biliary patency. APC, argon plasma coagulation; EUS, endoscopic ultrasound.Video 1

This case highlights that, when appropriately selected and combined, advanced endoscopic technologies can enable complex intraductal therapies and rescue situations previously considered unsalvageable.

Endoscopy_UCTN_Code_TTT_1AR_2AZ Endoscopy_UCTN_Code_TTT_1AR_2AG
